# TEAD transcription factor family emerges as a promising therapeutic target for oral squamous cell carcinoma

**DOI:** 10.3389/fimmu.2024.1480701

**Published:** 2024-10-04

**Authors:** Shuang Wang, Dan Shao, Xiaoyan Gao, Peng Zhao, Fanzhi Kong, Jiawei Deng, Lianzhu Yang, Wei Shang, Yaping Sun, Zhiguang Fu

**Affiliations:** ^1^ Department of Stomatology, Qingdao West Coast New District Central Hospital, Qingdao, China; ^2^ Department of Stomatology, Medical College of Qingdao Huanghai University, Qingdao, China; ^3^ Department of Oral and Maxillofacial Surgery, Qingdao Stomatological Hospital Affiliated to Qingdao University, Qingdao, China; ^4^ Department of Quality Inspection, Traditional Chinese Medical Hospital of Huangdao District, Qingdao, China; ^5^ Department of Stomatology, The Affiliated Hospital of Qingdao University, Qingdao, China; ^6^ Department of Tumor Radiotherapy, Air Force Medical Center, People's Liberation Army of China (PLA), Beijing, China

**Keywords:** TEA domain transcription factors, hippo signaling pathway, oral squamous cell carcinoma, molecular targeted therapy, biomarkers

## Abstract

The treatment of oral squamous cell carcinoma (OSCC) remains a significant difficulty, as there has been no improvement in survival rates over the past fifty years. Hence, exploration and confirmation of new dependable treatment targets and biomarkers is imperative for OSCC therapy. TEAD transcription factors are crucial for integrating and coordinating multiple signaling pathways that are essential for embryonic development, organ formation, and tissue homeostasis. In addition, by attaching to coactivators, TEAD modifies the expression of genes such as Cyr61, Myc, and connective tissue growth factor, hence facilitating tumor progression. Therefore, TEAD is regarded as an effective predictive biomarker due to its significant connection with clinical parameters in several malignant tumors, including OSCC. The efficacy of existing drugs that specifically target TEAD has demonstrated encouraging outcomes, indicating its potential as an optimal target for OSCC treatment. This review provides an overview of current targeted therapy strategies for OSCC by highlighting the transcription mechanism and involvement of TEAD in oncogenic signaling pathways. Finally, the feasibility of utilizing TEAD as an innovative approach to address OSCC and its potential clinical applications were analyzed and discussed.

## 1 Introduction

The TEAD (TEAD1-4) transcription factor family acts as the key terminal regulatory factor in the Hippo signaling pathway. They regulate cellular development, proliferation, and tissue homeostasis by modifying target genes. A recent study has demonstrated a strong correlation between the abnormal expression and activation of TEAD and the development and clinicopathological features of human malignant tumors, including oral squamous cell carcinoma (OSCC) (1–3). The expression of abnormal TEAD has been observed to impact important oncogenes, including MYC (4, 5), KRAS (6), BRAF (7), LKB1 (8), and NF2 (9, 10). The transcriptional activity of TEAD is crucial in multiple facets of tumor formation, encompassing tumor advancement, spread to other parts of the body, metabolic changes, immunological responses, and drug resistance mechanisms. Previous TEAD-related studies have predominantly concentrated on the examination of TEAD activity through the analysis of Hippo pathway kinases and YAP/TAZ proteins. However, recent findings have brought attention to the significance of post-translational alterations, intercommunication between carcinogenic signaling pathways, and nuclear localization dynamics as pivotal factors influencing TEAD activity. TEAD interacts with various transcription factors associated with signal transduction pathways, such as transcription factor (TCF) (11), activating protein 1 (AP-1) (12), MRTF (13), SMADs (14), and OCT4 (15), in addition to YAP/TAZ in the Hippo pathway. Recent research has found evidence that the disruption of the hippocampal signaling pathway is associated with various types of cancer, such as oral cancer, breast cancer, colorectal cancer, and cholangiocarcinoma (2, 16). A significant amount of research has been dedicated to studying therapeutic methods that focus on specifically targeting and locating the phosphorylation of YAP/TAZ within subcellular structures. Nevertheless, the inherent conformation of the YAP/TAZ protein structure is naturally unfolded, providing difficulties for potential drug development. Consequently, the TEAD protein presents itself as a potentially effective target for manipulating the Hippo pathway (17). In this review, a comprehensive analysis of recent progress in targeted therapy for OSCC was discussed with a particular focus on the transcriptional mechanism and involvement of TEAD in oncogenic signaling. Consequently, this study provides innovative viewpoints and a theoretical basis for future drug development and clinical implementation targeting TEAD.

## 2 The current status of targeted therapy for OSCC

OSCC is the most prevalent form of head and neck squamous cell carcinoma (HNSCC). It typically occurs in the lips, gums, tongue, oral cavity, and palate. OSCC significantly impacts human health and overall well-being, with a 5-year survival rate of around 50% (18, 19). The main clinical strategy for OSCC is a combination of surgery, radiation, and chemotherapy, which are determined based on the tumor’s stage and pathological diagnosis. Cisplatin (CDDP), paclitaxel, 5-fluorouracil, and doxorubicin are potential clinical medicines for OSCC in the form of monotherapy or combination (20). Nevertheless, some constraints, such as restricted therapeutic effectiveness, systemic toxicity, and drug resistance, have raised more and more attention during these conventional therapies (21).

The utilization of nanotechnology has facilitated the implementation of intelligent drug delivery systems (DDS) for targeted therapy in OSCC (22–24). Nanoparticles with particular physical and chemical characteristics, significantly improve therapeutic efficacy by providing precise administration of drugs and minimizing systemic drug exposure during oral cancer therapy (25, 26). These nanoparticles could be prepared by many forms of materials, including lipids, polymers, metals, and metal oxides (27–31). The unique characteristics of the microenvironment, including decreased pH levels, elevated concentrations of reactive oxygen species, and the excessive expression of certain enzymes and receptors, offer an alternative strategy for targeted therapy against OSCC (32). Therefore, there has been an application of a novel generation of tumor-responsive DDSs in the recent therapy of OSCC (33). Furthermore, as evidenced by the observed disparities between the tumor environment and normal tissues, stimulus-responsive DDS have demonstrated great potential in preclinical investigations (34), but are still hindered by the inherent instability and lack of control in the tumor microenvironment. Hence, the improvement of sensitivity and controllability of stimulus-responsive DDS is necessary to obtain more satisfactory clinical effects.

Active targeted therapy, which is different from passive targeted therapy, primarily targets specific receptors located on the surface of tumor cells, dramatically enhancing medication specificity by specifically supplying ligands on nanocarriers (35). Active targeted therapy has exhibited satisfactory feasibility for OSCC therapy, including gene therapy, immunotherapy, and biomimetic technologies (36). The utilization of gene therapy has attracted significant interest in the field of cancer for the treatment of hereditary and monogenic disorders (37). Gene therapy shows great potential in preventing the recurrence of OSCC by providing specific targets towards malignant regions (38). Most importantly, compared to traditional therapeutic modalities, gene therapy offers an accurate molecular-level OSCC treatment. However, before the application of gene therapy in clinical therapy, the exploration of suitable vectors is crucial for the effectiveness of transfection and successful introduction of the target gene.

Immunotherapy is a new strategy for treating OSCC that involves either blocking the immune system’s ability to detect cancer cells or boosting the immune response at the tumor site (39–42). Immunotherapy for oral cancer focuses on many immunological mechanisms and important checkpoints, including programmed death-1, and PD-L1 (36). However, the noticeable therapeutic advantages of OSCC have only been witnessed in a restricted subset of patients due to the diverse characteristics of patients, such as smoking and alcohol intake.

Much research has been dedicated to investigating changes in gene or protein expression within neoplastic cells through the identification of atypical genetic and epigenetic mutations. Previous research has shown that using specific molecular markers and receptors has great potential as a therapeutic strategy to selectively increase the expression of genes and direct targeted drug delivery to cancerous tissues (43). In recent times, the search for new biomarkers that may improve OSCC diagnosis, therapy, and prognosis has attracted a lot of attention. These biomarkers also hold promise for reducing negative effects and advancing precision medicine. Unfortunately, the available choices for treating patients with advanced oral cancer are still restricted, which puts forward the requirement for the exploration of novel therapeutic targets for OSCC.

## 3 TEAD family and characteristics

Transcription enhancer factors (TEFs), commonly referred to as TEAD transcription factors, were initially identified in 1987 (44). The nucleoproteins were first identified as positive trans-enhancer factors because of their capacity to bind to the overlap element of the SV40 enhancer B1 region. Later, it was shown that TEAD may bind to the human papillomavirus-16 (HPV-16) enhancer and activate the HPV-16 oncogenes E6/E7 (45). TEAD, as a highly conserved transcription factor, could be observed for its similarity in most eukaryotes (46). Mammalian species possess four TEAD genes: TEAD1 (also known as TEF-1/NTEF), TEAD2 (also known as TEF-4/ETF), TEAD3 (also known as TEF-5/ETFR-1), and TEAD4 (also known as TEF-3/ETFR-2/FR-19).

**Figure 1:**
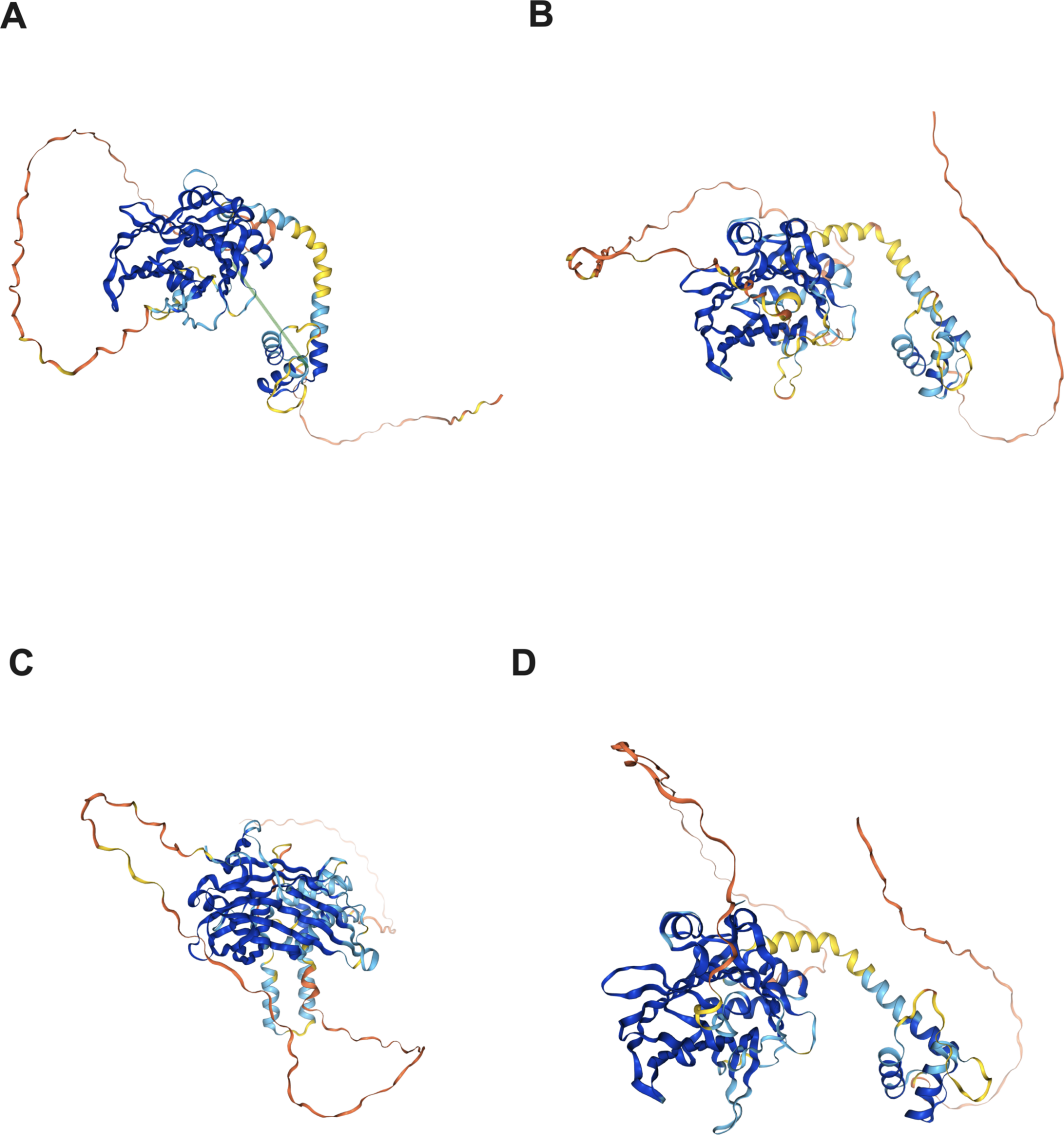
Protein structure of the TEAD family in the human protein atlas. **(A)** TEAD1 protein structure; **(B)** TEAD2 protein structure; **(C)** TEAD3 protein structure; **(D)** TEAD4 protein structure.

A notable similarity of the domain architecture could be observed in TEAD proteins, as depicted in **Figure 1**. The N-terminal TEA/ATTS domain of the TEAD protein folds similarly to other TEAD domains in order to interact with DNA. Additionally, a C-terminal deactivation domain that facilitates the recruitment of coactivators for binding to transcription target genes could also be observed (47). Notably, the level of similarity across human TEAD components in the DNA binding domain (DBD) exceeds 99%, endowing DBD to be the most conserved region within TEAD proteins (48). 5’-CATTCCA/T-3’, a highly conservative DNA binding site in the TEAD family, is commonly referred to as the MCAT element (49, 50). The C-terminal region, which contains the YAP binding domain (YBD), has been verified to be conserved (51). Linking to DNA is indicated by the location of the majority of TEADs within chromatin (52), but only minimal transcriptional activity could be provided by TEADs (53).

TEAD1, TEAD3, and TEAD4 display significant expression in multiple tissues, including pancreas, lung, heart, placenta, and others. In contrast, TEAD2 is selectively present in embryonic tissues such as the limbs, cerebellum, and testis but is largely absent in adult tissues (54). Although there is a significant similarity of human TEAD1-4, each TEAD protein has distinct expression in specific tissues, indicating the distinct function of each TEAD. Studies on gene inactivation have provided insights into the physiological roles of TEAD genes in mice. TEAD1 contributes significantly to the formation of the developing heart and enhances the expression of cardiac genes. Deletion mutations in TEAD1 result in embryonic death and defects in cardiac development (55). Nevertheless, there are divergent findings and controversies concerning the role of TEAD2. Kaneko et al. have documented that the deactivation of the TEAD2 gene substantially heightens the susceptibility to neural tube closure abnormalities in mice, commonly referred to as anencephaly (56). However, Sawada et al. reported that embryos with TEAD2 deletion were normal (57). The research conducted by Sawada et al. presented proof of the mutual participation of TEAD1 and TEAD2, as the mice with mutations in both TEAD1 and TEAD2 showed more prevalent malformations. TEAD3 has been demonstrated to exhibit specific expression in the placenta and various embryonic tissues (58, 59). Bone marrow genes and the development of trophectoderm are both regulated by TEAD4 (60, 61). Consequently, the inhibition of TEAD4 leads to the inability of the embryo to successfully implant into the endometrium (62). Additional research is necessary to examine the extent to which unique TEAD homologs complement one another in diverse physiological and carcinogenic contexts, as well as how their synergistic potential varies across different tissue types.

## 4 Regulation of TEAD transcription activity

The main regulatory mechanism of TEAD transcriptional activity is mainly related to binding coactivators. Notable transcriptional coactivators that impact TEAD function include Yes-associated protein (YAP), transcription coactivator with PDZ-binding motif (TAZ, also referred to as WWTR1), VgLL protein, and p160 protein (63).

YAP and TAZ (YAP/TAZ) are the targets and end-effectors of TEAD in the hippo pathway. They are found in the cytoplasm and are unable to bind to TEAD when LATS1/2, which is a large tumor suppressor kinase, is phosphorylated. This inhibits the activation of TEAD transcription. After being dephosphorylated, the YAP/TAZ complex is transported to the nucleus where it interacts with TEAD to promote the transcription of target genes that are essential for cellular growth, survival, and division. The inactivation of LATS1/2, dephosphorylation, and consequent aggregation of YAP/TAZ inside the nucleus are further caused by the down-regulation of the Hippo pathway. These aggregates then bind to TEAD and up-regulate the expression of specific genes, such as connective tissue growth factor (CTGF) and Cyr61 (64, 65). In conclusion, inhibition of the hippo pathway could achieve multiple biological behaviors, including the promotion of the movement of YAP/TAZ to the nucleus, binding with the TEAD transcription factor in the transcription enhancer-associated domain, stimulating the expression of target genes, and enhancing cell proliferation (66).

Furthermore, there are TEAD coactivators that are not associated with the hippo route. The Vestigial-like (VGLL) protein family includes four coactivators: VGLL1, VGLL2, VGLL3, and VGLL4. The regulation mechanism of VGLL on TEAD has been demonstrated to be related to gene expression. Because the binding sites on TEAD that the VGLL family proteins have overlap with the binding sites of YAP/TAZ, they compete with YAP/TAZ for TEAD binding, which inhibits the development of tumors (67). TEAD4 interacts directly with TCF4 through its TEA domain, contributing to the deactivation of TCF4 and the regulation of Wnt target genes (11). Previous studies have shown a clear and direct connection between AP-1 and TEAD. AP-1 is crucial for activating target genes that are important for the development and progression of tumors (12). Additionally, the interaction between steroid receptor coactivators belonging to the p160 family and TEAD has been confirmed (68). Other coactivators, including poly (adp-ribose) polymerase (69), serum response factor (70), myocyte enhancer factor 2 (71), and myc-associated factor X, have been found to be related to TEAD transcription (72). Nevertheless, recent studies have shown that the transcription activity of TEAD may be regulated by post-translational modifications and changes in its location within the cell.

S-palmitoylation is a type of post-translational modification that affects the activity of the TEAD family. This modification occurs independently of the binding of coactivators. The palmitoylation sites in TEAD have been identified as three conserved cysteine residues. Palmitoylation in TEAD is essential for controlling protein stability (73) and transcriptional activity (74). The S-palmitoylation of TEAD1 plays a crucial role in the binding and regulation of YAP/TAZ, and has significant biological significance. The structural changes in TEAD2 palmitoylation resulted in a significant decrease in the abundance of TEAD2 protein, primarily due to the reduction in protein stability (73). Palmitoylation is of specific significance in both protein transport and membrane localization (75), while TEAD palmitoylation does not impact its localization or binding to the membrane (73, 74). The results of structural investigations indicate that TEAD’s palmitoyl group is located inside a deep hydrophobic cavity. However, it is yet unknown if TEAD depalmitoylation mechanism can be controlled and if TEAD palmitoylation is a dynamic process.

Different from post-translational changes, transcription factors can be spatially regulated to modify transcriptional activity. Although studies on subcellular changes in TEAD localization are limited, the regulation mechanism of nuclear translocation of TEAD in target gene expression obtains more and more evidence. Recently, the influence of environmental stresses in TEAD subcellular localization has been demonstrated. The cytoplasmic localization of TEAD and its transcriptional inactivation are significantly increased by hypertonic stress, high cell density, and cell detachment, as reported in a study (76). The process of hypertonic stress-induced TEAD cytoplasmic localization is proven to be facilitated by p38, rather than the Hippo pathway. During extended osmotic stress, p38 binds directly to TEAD and moves TEAD to the cytoplasm, which leads to the suppression of TEAD transcription (77). Nevertheless, the specific biochemical process by which cell density triggers TEAD cytoplasmic localization has yet to be fully understood.

The subcellular location of TEAD has been demonstrated to exert a dominant influence on the regulatory signaling pathway of YAP/TAZ, hence impeding the growth of cancer cells driven by YAP (78). The translocation of YAP/TAZ into the cytoplasm under osmotic pressure is attributed to the translocation of TEAD. Furthermore, alterations in the subcellular location of TEAD can also contribute to the control of TEAD gene expression and determining the output of Hippo signaling.

## 5 TEAD-related pathways

### 5.1 Hippo signaling pathway

The Hippo pathway, a signaling pathway that has been conserved throughout evolution, is controlled by factors such as cell-to-cell contact, cell polarity, mechanical signals, g-protein-coupled receptor ligands, and cellular energy status (79, 80). The Hippo pathway is linked to various cellular processes such as development, proliferation, morphology, and growth of cells. It also plays a role in tissue regeneration, regulation of cancer immunity, innate immune responses, and autoimmune diseases (81–83). The Hippo pathway is implicated in key mechanisms that regulate aging, including the amp-activated protein kinase and sirtuin pathways, as well as autophagy and oxidative stress response/antioxidant defense. This suggests that targeted molecular therapies aimed at the Hippo pathway could be used to address aging and cancer more effectively (84).

In mammals, the Hippo signaling pathway was initially discovered in fruit flies and is known to suppress cell proliferation and stimulate apoptosis, thus inhibiting excessive growth of organs (85–88). The main members of the Hippo pathway in mammals include Ste20 family kinase MST (MST1/2), scaffolding protein Salvador/WW45 (SAV1), NDR family kinase LATS (LATS1/2), MOB kinase activator (MOB1A/B), Yes-associated protein YAP, transcription coactivator with PDZ-binding motif TAZ, and transcription factor TEAD1-4 (89). Merlin/NF2 proteins, known as the activators of the Hippo pathway, activate kinase cascade signaling pathways by producing cytoskeleton complexes (90, 91). The phosphorylation of downstream LATS1/2 is promoted by the activation of MST1/2 through its interaction with SAV1, thus initiating the kinase activity of the LATS1/2-MOB1 complex (92). This leads to the subsequent termination of YAP and TAZ activity through phosphorylation. YAP/TAZ activity is specifically hindered by phosphorylation, which creates a binding site on the 14-3-3 protein. This causes YAP and TAZ to be confined within the cytoplasm (93, 94). The final effector molecules of the Hippo pathway are YAP and TAZ, which are considered the essential core kinase components of the pathway (95). The YAP/TAZ protein undergoes dephosphorylation, leading to its migration from the cytoplasm to the nucleus, where it inhibits the Hippo pathway. The YAP/TAZ protein engages in interactions with various transcription regulators within the nucleus, including p73, p53BP2, RUNX2, SMAD7, ERBB4, PEBP2α, and TEAD, which is of big significance in regulating the transcription of downstream genes (96–100). Furthermore, by direct interaction with TEAD, YAP/TAZ protein is also involved in tumor growth and metastasis, such as cell proliferation, transformation, migration, and invasion (101).

### 5.2 The Wnt pathway

As a signal transduction pathway that activates multiple downstream channels, the Wnt pathway consists of various components, such as the Wnt protein (also known as Wnt ligand), the Wnt receptor (comprising Frizzled family protein and low-density lipoprotein receptor-associated protein LRP-5/6), the Dishevelled (Dsh/Dvl) protein, β-catenin, glycogen synthase kinase 3β, Axin/Conductin, and APC (adenomatous polyposis coli) protein (102). The Wnt/β-catenin pathway and the nonclassical Wnt pathway are two prominent upstream signaling members in the regulation of TEAD. The conventional Wnt/β-catenin pathway controls the expression of TEAD and YAP/TAZ genes through both hippo-dependent and non-dependent pathways (103). The nonclassical Wnt pathway, which operates separately from β-catenin and the degradation complex, plays a substantial role in carcinogenesis, differentiation, development, and the suppression of Wnt/β-catenin signaling. YAP/TAZ is essential for controlling various biological behaviors associated with nonclassical Wnt signaling through TEAD. These processes include gene expression, osteogenic differentiation, cell migration, and the inhibition of Wnt/β-catenin signaling (104).

Multiple interactions between Hippo and Wnt/β-catenin signaling have been demonstrated. Activation of the Hippo pathway triggers Hippo signaling, which results in the phosphorylation of serine/threonine residues on YAP1 and TAZ and further development of a complex that destroys β-catenin. On the other hand, the dephosphorylation of YAP1 or TAZ, along with the methylation of YAP1, induces the inactivation of the β-catenin destruction complex and the subsequent nuclear localization of β-catenin. During the “close” state of the Hippo pathway, YAP1 and TAZ attach to nuclear β-catenin and engage in β-catenin-dependent transcription activities (105). The convergence of the Wnt and Hippo pathways on TEAD is achieved through both degradation complex-dependent and independent mechanisms. These mechanisms have been extensively studied in the fields of cancer, stem cell biology, and development. The activity of TEAD is crucial for the biological responses induced by Wnt in various biological behaviors. The Wnt pathway promotes the activation of TEAD, which in turn suppresses Wnt signaling through its transcription outputs. This establishes a negative feedback mechanism. Studies have shown that the activation of the Wnt pathway leads to the activation of YAP/TAZ and TEAD, which in turn are associated with the advancement of breast cancer, resistance to chemotherapy, preservation of stem cells, and polarization of macrophages (105, 106). Although YAP has been demonstrated to inhibit Wnt-induced biological responses in tumors and stem cells, the precise function of TEAD transcription outputs necessitates additional research (107–109).

### 5.3 TGF-β pathway

The TGF-β pathway is responsible for controlling various biological processes, such as embryonic development, stem cell differentiation, immune regulation, wound healing, and inflammation. This pathway plays a crucial role in both mature organisms and developing embryos (110, 111). The initiation of the signaling pathway commences with the attachment of TGF-β family ligand molecules to the receptor, leading to the phosphorylation of TGF-β. Once phosphorylated, TGF-β I directly interacts with the substrate SMADs protein, which then transmits the signal from the cell membrane and cytoplasm to the nucleus. The activated SMADs collaborate with other nuclear factors to either stimulate or suppress the transcription of target genes (112).

As a crucial regulatory cytokine, TGF-β is involved in tumor suppression, invasion regulation, immune regulation, and regulation of the tumor cell microenvironment. TGF-β has a multifaceted bidirectional function in tumor tissue. During the initial stages of tumor development, it functions as a suppressor of epithelial cell growth, effectively preventing tumor growth; however, in advanced or late-stage tumors, it promotes tumor growth (113). Hence, the TGF-β response is significantly regulated by the specific circumstances during cancer development (114).

The interplay between TGF-β and the Hippo pathway is evident in the interaction between Smad and TEAD transcription factors. TGF-β triggers TEAD-mediated biological responses during cell differentiation, oncogenesis, and fibrosis, which can be influenced by SMADs or occur without their involvement. TGF-β promotes TEAD expression, which can also directly trigger TGFβ signaling conversely. The expression of TEAD target genes induced by TGFβ promotes the transition of breast epithelial cells from an epithelial to a mesenchymal state, known as epithelial-mesenchymal transition (EMT), and contributes to the development of malignant tumor characteristics (115). It is worth mentioning that the TGF-β-binding I ligand is directly regulated by TEAD, resulting in a positive feedback mechanism (116). TGF-β-induced oncogenesis is also mediated by TEAD. Inactivation of the Hippo pathway could be observed in most malignant mesotheliomas, in which the generation of the YAP-TEAD4-Smad3-p300 complex at the CTGF promoter is synergized with the TGFβ pathway (117). The TGFβ-YAP/TAZ-TEAD signaling pathway plays a critical role in late metastatic phenotypes in breast cancer cells (118). Furthermore, the biological significance of the TEAD transcription factor in TGFβ-pathway-related oncogenesis and development needs further investigation and discussion.

## 6 TEAD and tumors

### 6.1 TEAD and systemic tumors

TEAD transcription factors are known to be critical mediators of normal cell growth and tumorigenesis and have emerged as important catalysts for cancer development, tumor growth, EMT, metastasis, and drug resistance. They are also necessary for the development and promotion of various types of cancer (119). TEAD regulates several genes closely associated with tumorigenesis, such as CTGF and Cyr61 (64), AXL receptor tyrosine kinase (120), and mesothelin (121).

There is evidence that the TEAD family expression is upregulated in a variety of cancer types, including gastric cancer, colorectal cancer, liver cancer, lung cancer, breast cancer, fallopian tube cancer, ovarian cancer, germ cell cancer, prostate cancer, kidney cancer, medulloblastoma, skin cancer, melanoma, Kaposi’s sarcoma, and HNSCC (122–124). TEAD expression has been observed to decrease in specific instances of breast cancer and kidney cancer (63). However, it has also been demonstrated that up-regulation of TEAD expression was linked to unfavorable clinical outcomes and can serve as prognostic indicators for various types of solid tumors, such as breast cancer (125), colorectal cancer (126, 127), gastric cancer (128, 129), prostate cancer (130), HNSCC (131), and others. The meta-analysis demonstrates a strong correlation between YAP and TAZ and unfavorable overall survival and disease-free survival in multiple types of cancer. These findings indicate that TEAD and YAP/TAZ expression can be used to predict the development of various types of malignant tumors in patients (132, 133).

**Figure 2:**
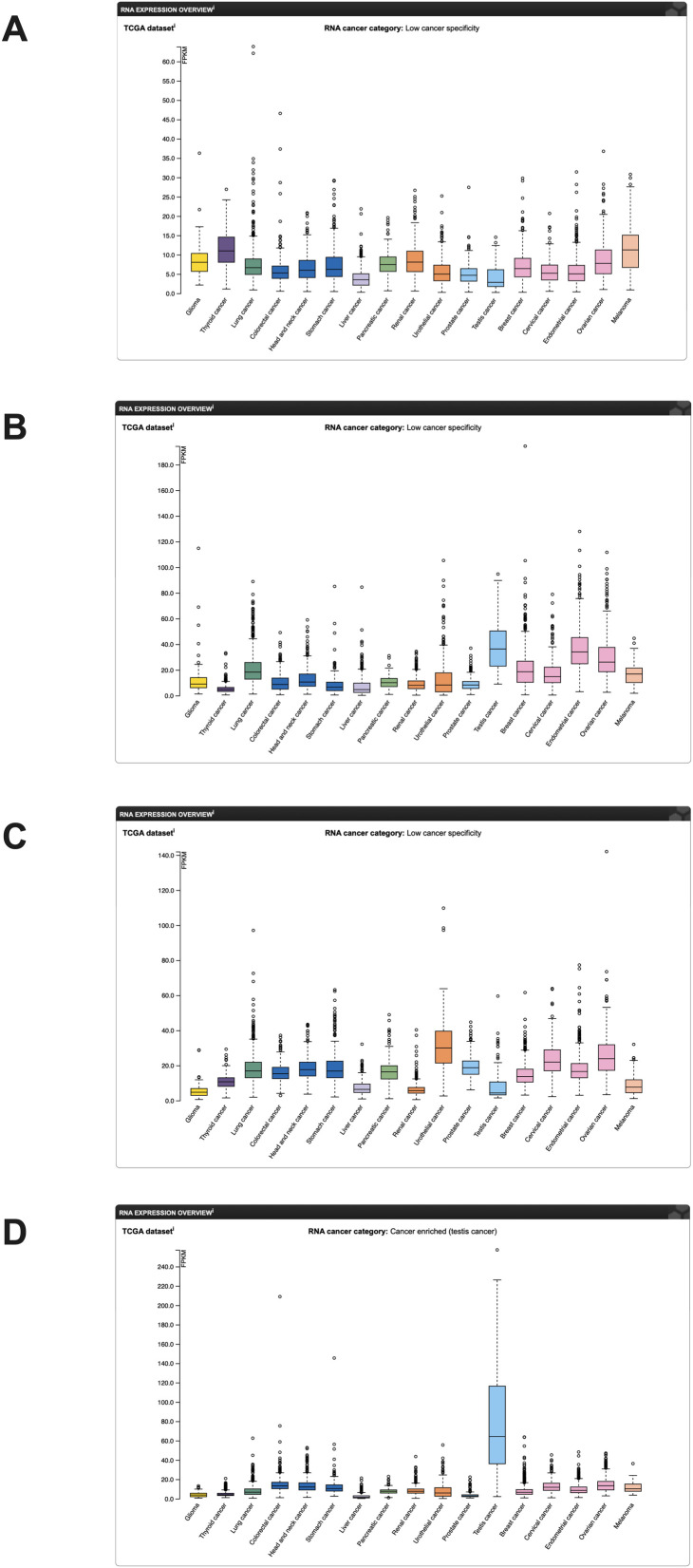
mRNA expression of the TEAD family in cancer in the human protein atlas (mRNA data from the TCGA database). **(A)** TEAD1 mRNA expression; **(B)** TEAD2 mRNA expression; **(C)** TEAD3 mRNA expression; **(D)** TEAD4 mRNA expression.

**Figure 3:**
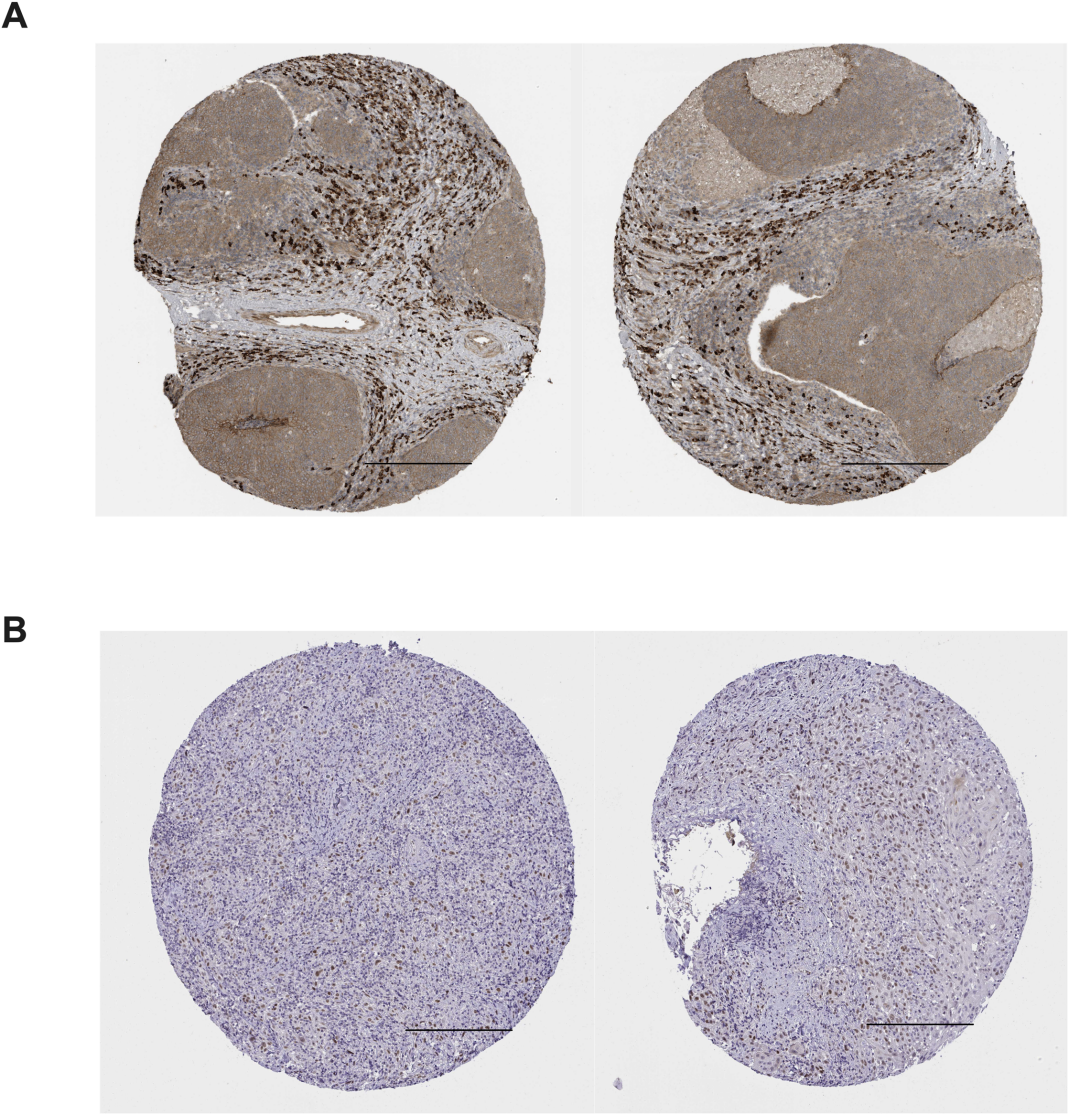
Immunohistochemical staining of TEAD3 and TEAD4 in HNSCC tissues in the human protein atlas. **(A)** positive expression of TEAD3 in HNSCC tissues; **(B)** positive expression of TEAD4 in HNSCC tissues; Scale bars =200 μm.

The RNA expression map of TEAD in different cancers was acquired from https://www.proteinatlas.org/. (**Figure 2**), while the RNA-seq data for 17 cancer types were obtained from the Cancer Genome Atlas (TCGA). Immunohistochemical analysis data from the human protein atlas revealed a significant upregulation of TEAD3 and TEAD4 in HNSCC cancer tissues (**Figure 3**). Unfortunately, the immunohistochemical data for TEAD1 and TEAD2 in cancer tissues were absent, necessitating further studies to confirm the protein expression of TEAD.

### 6.2 TEAD and OSCC

A positive correlation between elevated TEAD4 expression and tumor size in patients with OSCC has been demonstrated by case studies. The knockout of TEAD4 induced the cell cycle to be halted in the G1 phase, leading to a notable reduction in cell proliferation. It is plausible that the presence of the TEAD4-YAP complex within the nucleus is intricately linked to the transcription of genes associated with G1 inhibition. Deletion of TEAD4 resulted in elevated YAP phosphorylation and reduced YAP localization in the nucleus. Consequently, the expression of TEAD4 has a close relationship not only with the DNA binding and transcription activity of YAP but also with its phosphorylation (134). Research of TCGA data set analysis and clinical case determination revealed a significant association between increased TEAD4 expression and various negative outcomes, including a high pathological grade, the spread of cancer cells to cervical lymph nodes, an advanced stage of the disease, reduced overall survival, and a lower chance of being disease-free (131). This study provided a worthy analysis of the correlation between illness development and elevated TEAD4 immunostaining in a mouse model produced by 4nqs.

The gene silence of TEAD4 led to a significant suppression of the proliferation, migration, and invasion of CAL-27 cells, along with the initiation of apoptosis. Conversely, over-expression of the TEAD4 gene led to the opposite outcomes, confirming the significant role of TEAD4 in TGF-β1-induced EMT in CAL-27 cells.

Verduci et al. (135) examined samples from 115 patients with HNSCC, including 73 OSCC, and discovered that circPVT1 was overexpressed in tumors relative to matched non-tumor tissues and was especially prominent in individuals with TP53 mutations. CircumPVT1, as an oncogene, regulated the expression of miR-497-5p and other genes implicated in the modulation of cellular proliferation. The mut-p53/YAP/TEAD complex transcriptionally activated circPVT1, resulting in its up-regulation in CAL27 cells.

## 7 TEAD as a promising therapeutic target for OSCC

### 7.1 TEAD as a therapeutic target

Recently, there has been increased focus on the Hippo pathway as a potentially valuable target for therapy, as it plays a significant role in cellular proliferation and viability. Hippo pathway could be considered to be an anti-tumor therapeutic strategy because of the potential targeting of tumor suppressor factors Nf2, Mst1/2, and Lats1/2 by several members of the pathway, including the core kinase cascade. Nevertheless, there remains a deficiency in the availability of efficient techniques to stimulate these specific objectives. The most effective strategy for targeting the Hippo pathway involves focusing on transcription coactivators YAP and TAZ, in addition to TEADs (136). TEAD1-4 primarily consists of structural domains, in contrast to YAP/TAZ. There is a significant challenge for the development of TEAD antagonists due to the requirement of localization of the inhibitor to the nucleus and the high affinity and specificity of TEAD targeting. The conservation of TEAD1-4 and the limited understanding of the functional role of each member make it difficult to determine whether a specific inhibitor for a specific member or a broad-spectrum inhibitor for all human TEAD proteins is necessary.

#### 7.1.1 Targeting the TEAD DBD

There is still not enough work focusing on the selective DBD of TEAD as well as available inhibitors. In the initial demonstration of targeting DBD, it was observed that site-directed mutations in residues that bind to both small and large DNA channels could impede the proliferation of tumor cells (137). Because the DNA binding domain (DBD) shows a high degree of sequence conservation, inhibitors targeting this region would have broad specificity. Additionally, the intricate conformational and charge associations between DNA and protein pose considerable challenges in targeting transcription factors via their DNA-binding domains.

#### 7.1.2 Targeting TEAD liposomes

The hydrophobic pocket structure, known as a liposome, is formed in the YBD region of TEAD through palmitoylation modification following translation, and can be effectively targeted by small-molecule inhibitors (74). There have been reports indicating that liposomes containing TEAD2 and TEAD4 can bind together to regulate the Hippo pathway. The initial use of liposomes as therapeutic targets was discovered through a high-throughput screening method that utilized dynamic scanning fluorescence to identify ligands for stable TEAD4 YBD. Flufenamic acid (FA) and analogues were found from this initial screening to bind to the liposomes and inhibit the transcription of the TEAD-YAP-driven gene (138). While it has been demonstrated that molecules that target lipid sites can induce biological alterations in the TEAD transcriptome, the precise regulatory mechanism and crystal structure of the complex formed by FA and TEAD2 YBD are still not clear. For further comprehension of the application potential of lipid capsule antagonists in TEAD regulation and tumor therapy, more investigation on the mechanism and regulatory function of this post-translational modification at lipid sites is necessary (76).

In recent years, the self-palmitoylation of the TEADs protein family has emerged as a new type of post-translational modification, which has been identified through proteomics and chemical genomics. The feasibility of palmitoylation sites as highly suitable drug action sites with high targetability has been demonstrated by subsequent functional mechanism studies. However, the development of effective chemical and biological methods for screening TEAD palmitoylation sites is still hindered by the absence of TEAD palmitoylation-specific antibodies and the deep embedding of palmitoylation site pockets. Consequently, existing drug development efforts targeting this site need more promotion, and high-activity and high-specificity chemical probes are in urgent need to explore the related functional mechanisms.

#### 7.1.3 Targeting the TEAD-YAP interface

Currently, the most promising therapeutic approach for enhancing the transcriptional output of the Hippo pathway is to disrupt the TEAD-YAP interface. Initial findings from liver cancer models driven by YAP indicate that the development of normal tissues would not be hindered by overexpression of TEAD (139), which provides a possibility to design inhibitors that can maintain the TEAD-YAP complex’s equilibrium within the nucleus. In addition, the counteractive effect of coactivators Vgll and p160 also needs to be considered when designing inhibitors that specifically target the TEAD-YAP interface. Some promising compounds interfering with the TEAD-YAP interface, have been designed in previous research. The inhibition of TEAD in tumors by these small molecules and peptides has been demonstrated in both *in vitro* investigation and *in vivo* procedure. A recent study has shown that VGLL4 mimetic peptide therapy effectively hinders the growth of gastric cancer by binding to YAP-competitive TEAD (140). Verteporfin, a benzoporphyrin derivative, is used in clinical settings for photodynamic therapy to treat neovascular macular degeneration. It has been demonstrated to hinder the expression of specific genes in the Hippo pathway by disrupting the formation of the YAP-TEAD complex. This disruption leads to the inhibition of tumorigenesis, invasion, and angiogenesis (141). In addition, verteporfin may also hinder the growth of cells and retinoblastoma cells without photoactivation by inhibiting the YAP-tead complex. Verteporfin has been shown to inhibit YAP-induced tumor cell growth and invasion by down-regulating target genes in the Hippo signaling pathway. This effect has been confirmed in various types of cancer, including liver cancer (142), bladder cancer (143), gastric cancer (144), breast cancer (145), colon cancer (146), pancreatic cancer (147), ovarian cancer (148) and melanoma (149).

In another work, nucleic acid-targeting NLS18-TEAD was synthesized as a chimeric trifunctional peptide consisting of a cell-penetrating peptide, a nuclear localization sequence, and a disrupting peptide that blocks TEAD-YAP interaction. This study demonstrated that nuclear targeting, which depends on specific protein/protein interactions within the nucleus, shows great potential as a method for targeting the Hippo signaling pathway (150). However, the molecular framework of various aspects of the Hippo pathway, particularly the mechanisms by which the pathway is activated, inactivation, and transcriptional activation of the pathway, remains unclear. Therefore, it is crucial to investigate the efficiency and biosafety of inhibitors that block TEAD-YAP transcription. Elucidating Hippo biomarkers in clinical studies related to TEAD is crucial for evaluating the effectiveness of inhibitors that target the Hippo pathway. This is one of the several challenges that must be addressed before TEAD inhibitors can be used in clinical practice. While there is a scarcity of studies on the suitability of TEAD inhibitors for OSCC, these above findings were of big significance for the continuous research and treatment of OSCC.

### 7.2 TEAD and chemotherapy resistance

The disruption of the Hippo signaling pathway is a major contributing factor to the emergence of chemotherapy resistance in tumors. Elevated levels of YAP and TAZ have been demonstrated to be associated with chemotherapeutic resistance of regents such as cisplatin and cetuximab in OSCC (151–153). Activation of YAP/TAZ can enhance cell survival against chemotherapeutic drugs like 5-fluorouracil, cisplatin, and paclitaxel, thereby contributing to the development of different cancer types (154, 155). Activation of the YAP pathway also enhances susceptibility to targeted therapies, including tyrosine kinase, RAF, and MEK inhibitors. In contrast, reduced YAP levels increase the susceptibility to cisplatin and tyrosine kinase inhibitors like erlotinib and cetuximab (156–158). Bai et al. proposed that the chemosensitivity of liver cancer cells could be enhanced by p53 regulation of YAP, which requires the TEAD binding domain (159). TEAD induced the occurrence and drug resistance of esophageal cancer by directly attaching to the EGFR promoter to up-regulate EGFR expression (160). Another study showed that, MicroRNA-608 could sensitize cisplatin therapy of non-small cell lung cancer cells by targeting TEAD2, and overexpression of TEAD2 reduced miR-608-induced apoptosis of A549 cells under cisplatin (161).

Furthermore, the decrease in activity of the central kinase of the Hippo pathway is probably associated with the emergence of chemotherapy resistance of cancer cells (162). Cisplatin resistance of prostate cancer cells is promoted by the decrease in expression of MST1, which is controlled by heat shock protein 70 through a process that relies on the proteasome (163). A notable overexpression of miR-149-5p could be observed in chemotherapy-resistant ovarian cancer tissues and cell lines, which promotes the TEAD transcription, the nuclear translocation of YAP/TAZ, and the expression of many downstream genes within the Hippo pathway. The inhibition of the Hippo pathway by miR-149-5p has been observed to enhance treatment resistance in ovarian cancer cells towards CDDP (164). Overexpression of miR-181c in human pancreatic cancer cells led to excessive activation of YAP/TAZ, along with increased expression of hippo signaling downstream genes CTGF, BIRC5, and BLC2L1, which improved the *in vitro* and *in vivo* survival and resistance of pancreatic cancer cells to chemotherapy (165). The findings of this research indicate that, the down-regulation of the Hippo pathway, whether through the overexpression of YAP/TAZ and TEAD or the decreased expression of members of the Hippo pathway, is linked to the emergence of chemotherapy resistance in human cancer.

## 8 Conclusions and prospects

TEAD has been observed to be overexpressed in OSCC and other types of cancer. The involvement of TEAD in the process of carcinogenesis and the therapeutic advantages of TEAD targeting have gotten much attention based on experimental findings and emerging data. Although significant advancements in comprehending the hippocampus route, there are still unsolved inquiries in OSCC. Further *in vivo* validation is necessary in order to substantiate our existing *in vitro* findings. The investigation of TEAD expression in OSCC and its oncogenic mechanism is currently underway.

There are two crucial matters that require attention in the upcoming research. The initial aspect pertains to the intricate sequence of TEADs implicated in the advancement of tumors, encompassing tumor formation, EMT, resistance to drugs, metastasis, and many molecular mechanisms and activities. Despite an increasing understanding of the carcinogenic role of the TEAD transcription factor, the specific mutation responsible for driving carcinogenesis remains unclear. The expression and activation of TEADs may be determined by abnormal DNA copy number, transcriptional regulation, post-transcriptional regulation of miRNA, and post-translational modification of subcellular localization. A comprehensive analysis and examination of TEAD transcription co-activators will provide a clearer understanding of the dual function of TEADs in both normal and abnormal physiological processes. Furthermore, there remains a need for further development and research on medications targeting TEADs. Although various small-molecule TEAD inhibitors have demonstrated promise anti-tumor effects in preclinical research and are in clinical trials, inhibitors with high selectivity and low nephrotoxicity remain to be discovered. Given that TEAD plays a significant part in important pathways related to tumor progression, further progress in comprehending the regulatory mechanisms of TEAD and creating therapeutic interventions will create a stimulating new area for fundamental scientific research and pharmaceutical development.
